# A case of bilateral hilar and mediastinal lymphadenopathy developing during treatment for *Mycobacterium avium* complex

**DOI:** 10.1002/ccr3.1024

**Published:** 2017-06-01

**Authors:** Yumie Yamanaka, Akimasa Sekine, Hideaki Yamakawa, Tomohisa Baba, Koji Okudela, Tamiko Takemura, Takashi Ogura

**Affiliations:** ^1^ Department of Respiratory MedicineKanagawa Cardiovascular and Respiratory CenterYokohamaJapan; ^2^ Department of Respiratory MedicineTokyo Jikei University HospitalTokyoJapan; ^3^ Department of PathologyGraduate School of MedicineYokohama City UniversityYokohamaJapan; ^4^ Department of PathologyJapan Red Cross Medical CenterTokyoJapan

**Keywords:** Bilateral hilar lymphadenopathy, *Mycobacterium avium* complex, *Propionibacterium acnes*, Sarcoidosis

## Abstract

We report a rare case of an immunocompetent patient with *Mycobacterium avium* complex (MAC) disease in which bilateral hilar lymphadenopathy developed during anti‐MAC treatment. This case indicates that *Propionibacterium acnes* would be present and might be a cause of sarcoidosis even in patients with MAC.

## Introduction

Sarcoidosis is a multiple organ system disorder of unknown etiology, characterized by noncaseating granulomatous tissue infiltration and inflammation 1. In total 50–80% of sarcoidosis patients present with bilateral hilar lymphadenopathy (BHL), which is mostly detected at health examinations 2. Sarcoidosis has been reported to be potentially caused by mycobacterial organisms such as *Mycobacterium tuberculosis* and *Mycobacterium avium* complex (MAC), and *Propionibacterium acnes* (*P. acnes*) 1, 3. Here, we report a case of MAC in which BHL developed during anti‐MAC treatment.

## Case Report

**Figure 1:**
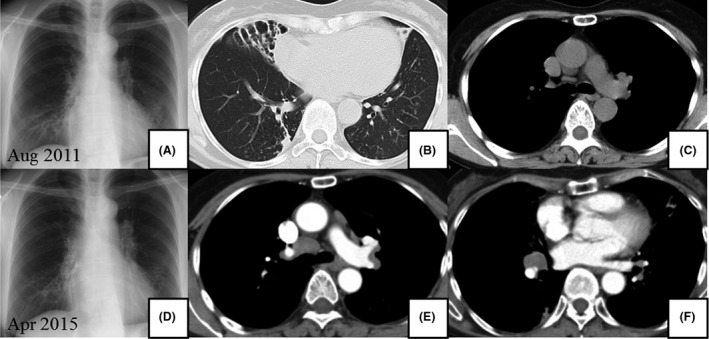
(A–C) Chest X‐ray and CT on the first visit revealed bronchiectasis and centrilobular small nodules. (D–F) Chest X‐ray and CT in April 2015 revealed bilateral hilar and mediastinal lymphadenopathy, particularly in the area of the middle lobe.

**Figure 2:**
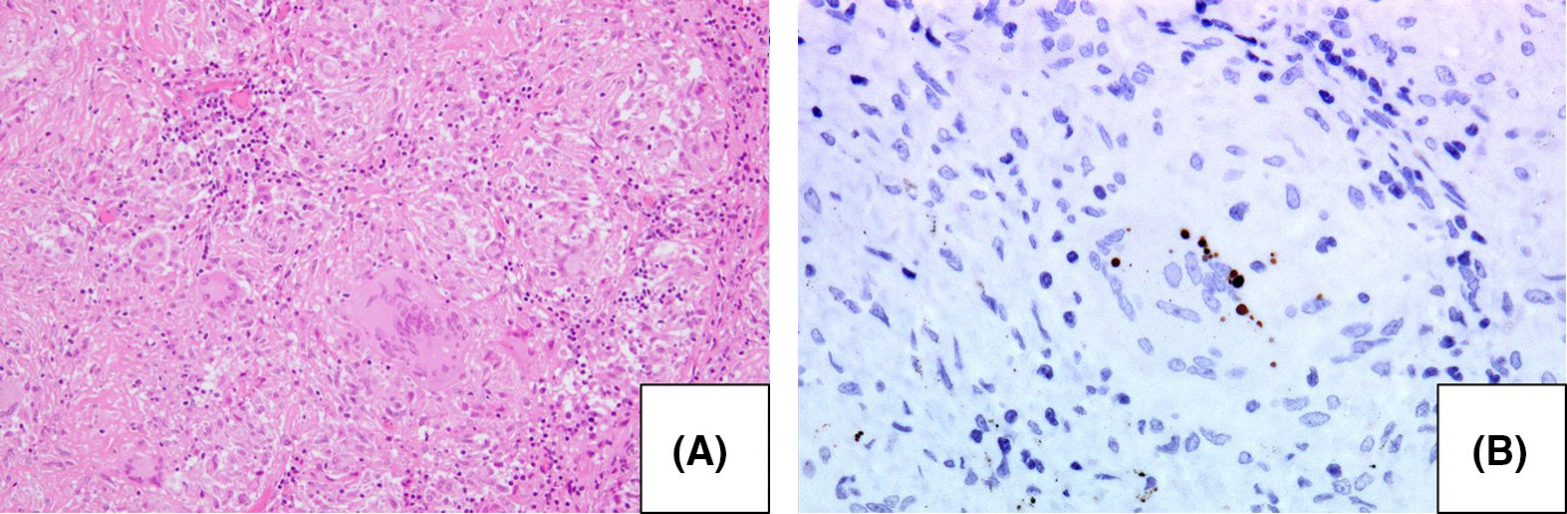
(A) Lymph node biopsy revealed many noncaseating granulomas (open arrow) (hematoxylin and eosin staining). (B) Small round bodies (closed arrow) were detected in the lymph node (immunostaining with Propionibacterium acnes‐specific monoclonal antibody).

**Figure 3:**
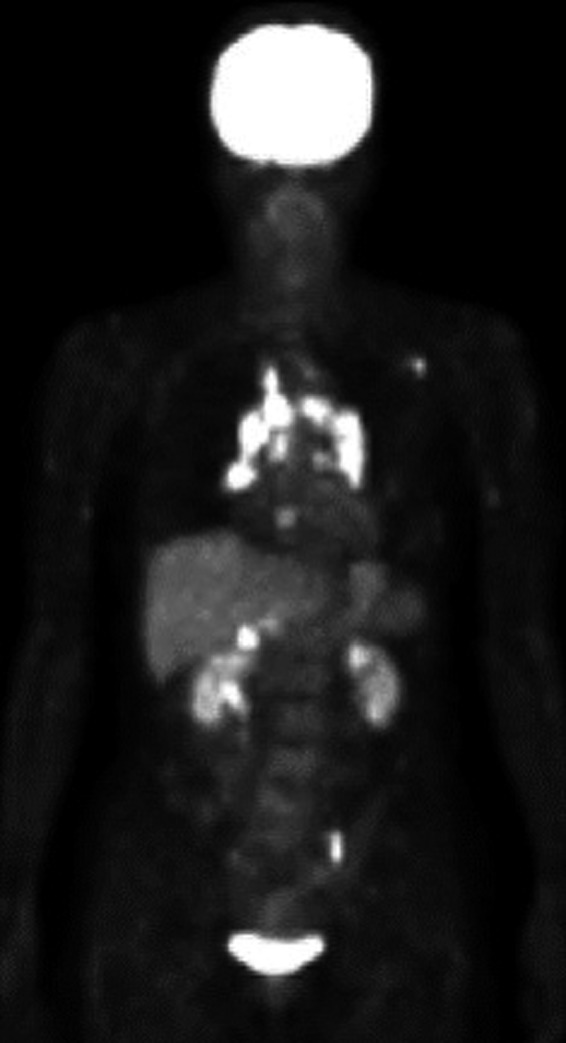
Fluorodeoxyglucose positron emission tomography revealed abnormal uptake in the bilateral hilar and mediastinal lymph nodes, left axillary lymph node, hepatic portal region lymph node, and left iliac artery region lymph node.

In November 2008, a 63‐year‐old woman who had never smoked presented to the hospital due to an abnormal chest shadow. She had previous medical histories of ovarian cysts and hepatitis B and was diagnosed with MAC lung disease by sputum culture and radiological feature. In August 2011, she visited our hospital because of bloody sputum, and chest X‐ray and computed tomography (CT) showed bronchiectasis and nodules in the left superior segment and right middle and lower lobes without an apparent lymphadenopathy (Fig. 1A–C). In August 2013, multidrug treatment with rifampicin (300 mg/day), ethambutol (500 mg/day), and clarithromycin (CAM, 800 mg/day) was started because chest CT revealed exacerbation in the superior segment of the left lung. The sputum smear and culture continued positive after 20‐month treatment, although the isolate was susceptible to CAM with no aggravation of the image. In April 2015, follow‐up chest X‐ray and CT showed the development of BHL and mediastinal lymphadenopathy (Fig. 1D–F). The lymphadenopathy was particularly evident in the right hilar lymph node. Although endobronchial ultrasound‐guided transbronchial needle biopsy was performed, the diagnosis was not confirmed. Because purulent excretion was bronchoscopically observed in the right middle lobar bronchus, and the right middle lobe was considered to be the main source of the spread of MAC via the airway. To reduce the MAC load and clarify the cause of BHL, right middle lobectomy and the right hilar lymph node biopsy were performed in July 2015. The specimen taken from the lymph node revealed noncaseous granulomas and some small round bodies which were immunohistochemically positive for *P. acnes*‐specific monoclonal antibodies (PAB antibodies) (Fig. 2), although mycobacterial organisms were not found bacteriologically or immunohistochemically. On the other hand, the resected lung was bacteriologically and immunohistochemically positive for mycobacteria and negative for *P. acnes*. In addition, fluorodeoxyglucose positron emission tomography (FDG‐PET) demonstrated the abnormal uptake in the bilateral hilar, the mediastinal, the left axillary, the portal, and the left common iliac lymph nodes (Fig. 3). Although the results of cardiac ultrasound, ophthalmological examination, angiotensin‐converting enzyme (18.4 IU/mL) level, and lysozyme (7.9 *μ*g/mL) level were normal, the pathological findings and the FDG uptake in systemic lymph nodes met the diagnostic criteria of sarcoidosis. After the lung resection, multidrug treatment was continued for 18 months without treatment for sarcoidosis, such as immunosuppressant agent including steroid. Although the sputum smear became negative, the size of BHL and mediastinal lymphadenopathy did not decrease.

## Discussion

In this report, we present a case of MAC lung disease in which BHL developed during anti‐MAC treatment. The clinical course of our patient has the following two clinical implications.

First, patients with MAC can develop BHL during anti‐MAC treatment. To the best of our knowledge, there is only one published case in which a patient with MAC developed BHL during anti‐MAC treatment 4. However, this case was complicated by disseminated MAC disease as a result of human immunodeficiency virus (HIV) infection, and BHL was considered to develop as an immune reconstitution inflammatory syndrome following anti‐HIV treatment. In our case, there was no underlying disease causing disseminated MAC disease. Considering the results of FDG‐PET and the noncaseous granulomas in the lymph node, it would be adequate that the systemic lymphadenopathy in our case developed as a manifestation of sarcoidosis. In addition, it could be though that even in the right hilar lymphadenopathy caused by sarcoidosis than MAC, because the lymph node was negative for mycobacterial organisms, and did not improve when the activity of MAC decreased.

Second, *P. acnes* might be a cause of sarcoidosis even in patients with MAC. The potential etiological agents of sarcoidosis are considered to be mycobacterial organisms, primarily *M. tuberculosis* and *P. acnes* 1, 5. The mechanism for *P. acnes* to trigger sarcoidosis was advocated that latent infection of *P. acnes* in lungs and hilar lymph nodes was endogenously activated by some kinds of environmental factors, which caused Th1 immune reaction in patients having the allergy factor for *P. acnes* as a host factor 6. Although *P. acnes* is common bacterium in lung tissue and mediastinal lymph nodes 7, *P. acnes* was reported to be frequently and specifically detected by the PAB antibody in sarcoid granulomas, but was not in nonsarcoid granulomas 8. Before performing lymph node biopsy, we had predicted that BHL was caused by MAC infection. However, the right hilar lymph node was bacteriologically and pathologically negative for MAC, but immunohistochemically positive for *P. acnes*. These results strongly indicate that *P. acnes* can cause lymphadenopathy, even in patients with MAC. On the other hand, the important thing is that lymphadenopathy was mostly evident in the right hilar lymph node, which was the main lesion of MAC infection. Although it remains unknown what mechanism facilitate to lymphadenopathy in our patients, MAC organism might contribute to development of lymphadenopathy caused by sarcoidosis. The previous paper by Hanngren A et al. demonstrated that a mycobacterial constituent was present in lymph nodes of sarcoidosis patients 9. *M. avium subspecies paratuberculosis* was also reported as a pathogen of Crohn's disease, which is a granulomatous disease 10. Importantly, a mannan expressed in *M. avium subspecies paratuberculosis* has been reported to impair the ability of monocytes and macrophages to kill phagocytosed‐*E. coli*, which would contribute to pathogenesis of Crohn's disease 11. As in Crohn's disease, MAC organisms might suppress phagocyte function and contribute to sarcoidosis development.

In conclusion, we report a rare case of an immunocompetent patient with MAC in which BHL developed during anti‐MAC treatment. This case would support the previous literature that *P. acnes* may be a cause of sarcoidosis, even in patients with MAC.

## Authorship

YY: wrote the initial draft of the manuscript. AS: contributed to analysis and interpretation of data, and assisted in the preparation of the manuscript. All other authors have contributed to data collection and interpretation, and critically reviewed the manuscript. The final version of the manuscript was approved by all authors.

## Conflict of Interest

The authors state that they have no conflicts of interest.
